# Identification, analysis, and linkage mapping of expressed sequence tags from the Australian sheep blowfly

**DOI:** 10.1186/1471-2164-12-406

**Published:** 2011-08-10

**Authors:** Siu F Lee, Zhenzhong Chen, Annette McGrath, Robert T Good, Philip Batterham

**Affiliations:** 1 Centre for Environmental Stress and Adaptation Research, Bio21 Institute, Genetics Department, University of Melbourne, 30 Flemington Road, Parkville, VIC 3010, Australia; 2 Australian Genome Research Facility, Level 5 Gehrmann Laboratories, University of Queensland, Research Road, St Lucia, QLD 4072, Australia

## Abstract

**Background:**

The Australian sheep blowfly *Lucilia cuprina *(Wiedemann) (Diptera: Calliphoridae) is a destructive pest of the sheep, a model organism for insecticide resistance research, and a valuable tool for medical and forensic professionals. However, genomic information on *L. cuprina *is still sparse.

**Results:**

We report here the construction of an embryonic and 2 larval cDNA libraries for *L. cuprina*. A total of 29,816 expressed sequence tags (ESTs) were obtained and assembled into 7,464 unique clusters. The sequence collection captures a great diversity of genes, including those related to insecticide resistance (e.g., 12 cytochrome P450s, 2 glutathione S transferases, and 6 esterases). Compared to *Drosophila melanogaster*, codon preference is different in 13 of the 18 amino acids encoded by redundant codons, reflecting the lower overall GC content in *L. cuprina*. In addition, we demonstrated that the ESTs could be converted into informative gene markers by capitalizing on the known gene structures in the model organism *D. melanogaster*. We successfully assigned 41 genes to their respective chromosomes in *L. cuprina*. The relative locations of these loci revealed high but incomplete chromosomal synteny between *L. cuprina *and *D. melanogaster*.

**Conclusions:**

Our results represent the first major transcriptomic undertaking in *L. cuprina*. These new genetic resources could be useful for the blowfly and insect research community.

## Background

The Australian sheep blowfly *Lucilia cuprina *(Wiedemann) (Diptera: Calliphoridae) is an important biological tool for medical treatment and forensic investigation. Disinfected blowfly larvae are routinely used in maggot debridement therapy to promote wound healing [1-3]. The necrophagous nature of *L. cuprina *also makes it invaluable for forensic analysis, particularly in estimating postmortem interval [4]. In contrast to these beneficial roles, *L. cuprina *is the primary cause of flystrike in Australia and New Zealand [5,6]. The practice of surgical mulesing, as well as various chemical insecticides, has been used to control this formidable pest. However, fly populations often evolved resistance rapidly [7-10]. Research into the genetic and biochemical mechanisms of resistance has provided some of the best examples of genetic adaptation to selection [11-13].

Despite its medical and agronomical importance and its historical status as one of the model organisms in insecticide resistance research, genomic information on *L. cuprina *is still relatively sparse. The haploid genome is approximately 810 mega bases [14], which is about 5 times the genome size of *Drosophila melanogaster*. Polytene *in situ *hybridization and genetic mapping studies have determined the basic organization of its 6 chromosomes [15-17]. In 1993, Weller and Foster published a recombination map based on 72 morphological and enzyme markers, and this remains the most comprehensive linkage map of *L. cuprina *to date [18]. The chromosomal location of these markers indicates that the major linkage elements (i.e., Muller's Elements) remain relatively conserved in higher Diptera [18].

To combat this insect pest more intelligently, it is desirable to improve our knowledge of its genetic makeup. Molecular tools have become increasingly accessible to generate large amount of information in a cost-effective manner. As the per-base cost of DNA sequencing continues to fall, large-scale expressed sequence tag (EST) projects have been accomplished in many insect species, and thousands of ESTs have been deposited in public databases, including higher dipteran species such as the screwworm *Cochliomyia hominivorax *[19] and the tsetse fly *Glossina morsitans *[20].

This paper describes a similar gene discovery effort to identify transcripts expressed in preadult stages (embryonic and larval). An assembly of 7,464 unique gene clusters was produced from a total of 29,816 ESTs. The protein-coding contents of this non-redundant dataset were evaluated via a series of homology analyses. We short-listed a subset of these *L. cuprina *genes, which showed high sequence conservation, favorable gene structure (suitable exon/intron positions and sizes), and single correspondence in the *D. melanogaster *and *Anopheles gambiae *genomes. To demonstrate their usefulness in comparative mapping, we carried out chromosomal assignment of 41 genes to infer inter-chromosomal rearrangements. Comparison between *L. cuprina *and *D. melanogaster *revealed a high but incomplete chromosomal synteny. This newly generated EST dataset is a significant step in the systematic buildup of genomic resources for this important insect in agricultural and medical entomology.

## Results

### Characteristics of EST assembly

**Figure 1:**
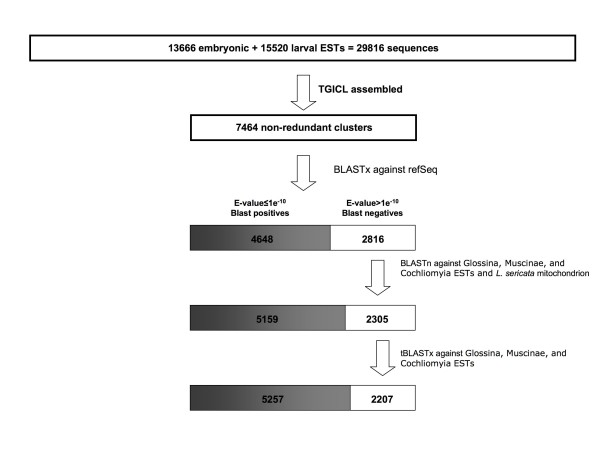
**An overview of the acquisition, assembly, analysis, and application *of L. cuprina*-expressed sequence tags**. A total of 29,816 ESTs from embryonic and larval libraries was assembled into 7,464 unique sequence clusters using the TGICL procedures. E-values from BLAST searches were arranged in ascending order from left to right, indicated by the darkness of shade.

**Figure 2:**
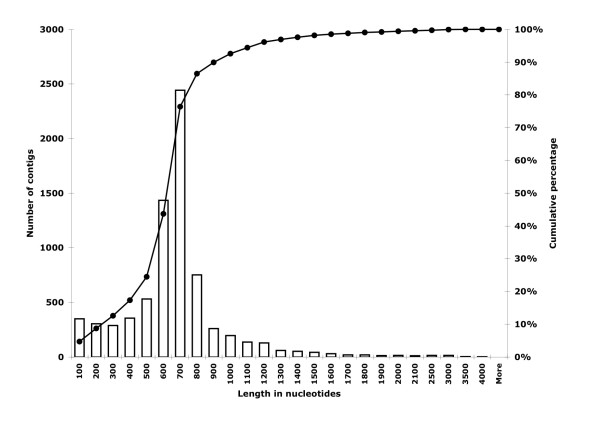
**Size distribution of the 7,464 non-redundant sequence clusters**.

**Figure 3:**
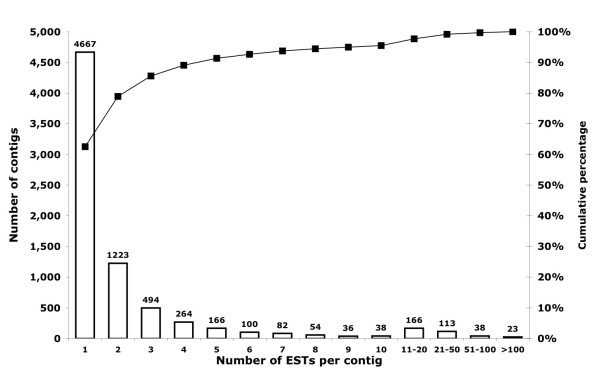
**Frequency distribution of contig sizes**.

We obtained 13,666 embryonic (JG422424-JG436089), 14,640 first-instar (JG407784-JG422423), and 880 third-instar (JG406904-JG407783) ESTs. The combined dataset (29,186 ESTs) was assembled into 7,464 unique clusters (Additional file 1), comprising 2,797 contigs and 4,667 singletons (Figure 1). The basic features of the sequence assembly are summarized in Figures 2 and 3. The majority (65.5%) of the 7,464 sequences were 500-900 bases in length (Figure 2), and the number of EST reads in a contig ranged between 2 and 3,700 (Figure 3), with an average of 8.8 reads (median = 3) per contig.

### Abundant transcripts in embryonic and larval stages

Since the cDNA libraries were not normalized, the number of reads in contigs can be used as an indication of levels of gene expression. Table 1 summarizes information about the most abundantly expressed genes, defined as contigs containing more than 100 ESTs. The mitochondria-derived transcripts dominate this list, accounting for a total of 5,275 ESTs, or 17% of the entire EST collection. In comparison to the published *Lucilia sericata *mitochondrial genome [21], our ESTs captured 12 protein coding genes and the 16S rRNA gene (Additional file 2). In addition to mitochondrial transcripts, various ribosomal protein genes (*RpL6, RpL7A, RpL7, RpL4*, and *RpS3A*), *18S rRNA*, and elongation factors (*ef1-α, ef2*, and *ef1-γ*) were also amongst the most highly expressed genes (Table 1), reflecting the robust translation and protein synthesis processes in the embryonic and larval stages.

**Table 1:** The most highly expressed genes in the EST dataset, indicated by the number of ESTs in a contig

**Contig name**	**Contig length (nt)**	**No. of EST reads in contig**	**Description**
**lucilia_CL1Contig5**	1972	3700	Mitochondrial 16S rRNA and 12S rRNA
**lucilia_CL4Contig1**	1560	679	Mitochondrial COI gene for cytochrome oxidase I and COII gene for cytochrome oxidase II
**lucilia_CL3Contig2**	2845	382	Elongation factor 1-alpha
**lucilia_CL3Contig3**	995	382	Ribosomal protein L6 (RpL6)
**lucilia_CL6Contig3**	878	258	Mitochondrial cytochrome-c oxidase subunit III
**lucilia_CL7Contig2**	1272	253	Mitochondrial cytochrome b
**lucilia_CL5Contig3**	3993	179	18S ribosomal RNA gene
**lucilia_CL2Contig20**	1065	168	Myosin regulatory light chain 2
**lucilia_CL10Contig1**	1804	163	Tubulin alpha-1 chain
**lucilia_CL5Contig4**	2551	158	Heat shock 70 kDa protein cognate 4
**lucilia_CL11Contig1**	1187	157	Ribosomal protein L7a (RpL7A)
**lucilia_CL3Contig8**	2596	153	Mitochondrial ATP synthase alpha subunit
**lucilia_CL12Contig1**	1214	133	ADP/ATP translocase
**lucilia_CL13Contig2**	2815	125	Arc1-like zinc binding protein (nucleic acid binding)
**lucilia_CL2Contig7**	2905	122	Elongation factor 2
**lucilia_CL2Contig49**	763	119	Mitochondrial ATP synthase lipid-binding protein
**lucilia_CL2Contig52**	2285	114	ATP-dependent RNA helicase p62 (nucleic acid binding)
**lucilia_CL17Contig1**	742	113	Mitochondrial COI gene for cytochrome oxidase I and COII gene for cytochrome oxidase II
**lucilia_CL16Contig1**	1199	112	Ribosomal protein L7 (RpL7)
**lucilia_CL14Contig2**	1780	110	Elongation factor 1-gamma
**lucilia_CL20Contig1**	1472	110	Ribosomal protein L4 (RpL4)
**lucilia_CL21Contig1**	1095	107	Ribosomal protein S3a (RpS3A)
**lucilia_CL19Contig2**	1124	103	Translationally controlled tumor protein

### GC content and codon usage bias

Based on results from a set of 200 conserved genes (Additional file 3), the average GC content (mean ± standard deviation) per coding sequence (CDS) is 0.4344 ± 0.0433 in *L. cuprina *and 0.5654 ± 0.0418 in *D. melanogaster*. The effective number of codons (Nc) is 43.81 in *L. cuprina *and 40.89 in *D. melanogaster*. Compared to *D. melanogaster, L. cuprina *shows a different codon preference for 13 of the 18 amino acids encoded by redundant codons (Table 2). The most noticeable changes occur in the preferred codons for glutamine (Q), glutamic acid (E), and leucine (L).

**Table 2:** Codon usage comparison between *L. cuprina *and *D. melanogaster *based on 200 conserved genes

		*L. cuprina*		*D. melanogaster*	
Amino acid	Codon	Fraction	Number	Fraction	Number
**Ala (A)**	GCA	0.094	350	0.088	337
**Ala (A)**	GCC	0.398	1480	0.602	2308
**Ala (A)**	GCG	0.021	77	0.114	436
**Ala (A)**	GCT	**0.487**	1812	0.196	751
**Cys (C)**	TGC	0.514	390	0.831	582
**Cys (C)**	TGT	0.486	369	0.169	118
**Asp (D)**	GAC	0.264	681	0.533	1371
**Asp (D)**	GAT	**0.736**	1897	0.467	1200
**Glu (E)**	GAA	**0.791**	2582	0.202	652
**Glu (E)**	GAG	0.209	684	0.798	2581
**Phe (F)**	TTC	0.62	1090	0.814	1429
**Phe (F)**	TTT	0.38	667	0.186	327
**Gly (G)**	GGA	0.121	407	0.223	742
**Gly (G)**	GGC	0.253	850	0.496	1652
**Gly (G)**	GGG	0.015	49	0.031	102
**Gly (G)**	GGT	**0.611**	2050	0.251	837
**His (H)**	CAC	0.485	458	0.689	648
**His (H)**	CAT	**0.515**	486	0.311	293
**Ile (I)**	ATA	0.123	335	0.073	192
**Ile (I)**	ATC	0.333	903	0.644	1692
**Ile (I)**	ATT	**0.544**	1475	0.283	742
**Lys (K)**	AAA	**0.518**	2101	0.147	572
**Lys (K)**	AAG	0.482	1958	0.853	3316
**Leu (L)**	CTA	0.049	194	0.043	170
**Leu (L)**	CTC	0.075	297	0.165	661
**Leu (L)**	CTG	0.038	149	0.552	2208
**Leu (L)**	CTT	0.113	445	0.07	281
**Leu (L)**	TTA	0.167	657	0.02	79
**Leu (L)**	TTG	**0.558**	2195	0.15	600
**Met (M)**	ATG	1	1208	1	1092
**Asn (N)**	AAC	0.431	993	0.738	1538
**Asn (N)**	AAT	**0.569**	1311	0.262	546
**Pro (P)**	CCA	0.277	563	0.185	375
**Pro (P)**	CCC	0.451	916	0.499	1013
**Pro (P)**	CCG	0.031	64	0.226	460
**Pro (P)**	CCT	0.241	490	0.09	183
**Gln (Q)**	CAA	**0.801**	1529	0.178	346
**Gln (Q)**	CAG	0.199	381	0.822	1595
**Arg (R)**	AGA	0.145	386	0.044	120
**Arg (R)**	AGG	0.028	74	0.074	200
**Arg (R)**	CGA	0.033	89	0.064	172
**Arg (R)**	CGC	0.223	595	0.48	1297
**Arg (R)**	CGG	0.009	24	0.08	217
**Arg (R)**	CGT	**0.562**	1498	0.258	696
**Ser (S)**	AGC	0.117	312	0.219	593
**Ser (S)**	AGT	0.141	376	0.062	168
**Ser (S)**	TCA	0.152	405	0.05	135
**Ser (S)**	TCC	0.253	675	0.343	929
**Ser (S)**	TCG	0.089	238	0.234	635
**Ser (S)**	TCT	0.247	659	0.093	251
**Thr (T)**	ACA	0.201	493	0.107	259
**Thr (T)**	ACC	0.431	1055	0.587	1421
**Thr (T)**	ACG	0.03	74	0.175	424
**Thr (T)**	ACT	0.337	825	0.131	316
**Val (V)**	GTA	0.218	733	0.052	174
**Val (V)**	GTC	0.24	806	0.31	1046
**Val (V)**	GTG	0.126	424	0.491	1657
**Val (V)**	GTT	**0.416**	1400	0.147	495
**Trp (W)**	TGG	1	430	1	438
**Tyr (Y)**	TAC	0.455	666	0.771	1099
**Tyr (Y)**	TAT	**0.545**	799	0.229	326
**STOP**	TAA	0.785	157	0.6	120
**STOP**	TAG	0.125	25	0.31	62
**STOP**	TGA	0.09	18	0.09	18
**Total**			47579		47095

The preferred codons for each amino acid are in underlined in each species; cases where the preferred codons in *L. cuprina *are different from those of *D. melanogaster *are indicated in bold.

### Protein-coding contents of EST assembly

To evaluate the protein coding contents of our ESTs, the 7,464 non-redundant sequences were subject to various homology searches against existing sequences (Figure 1). Our homology analyses showed that 5,257 (70%) of the non-redundant sequences had significant (E-value ≤ 1e^-10^) matches in the public domains (Additional file 4). The sequences that had recognizable homologs constituted 937 InterProScan and 494 Gene Ontology terms (Additional files 5 and 6), indicating that a great diversity of protein motifs and biological processes was represented in our dataset. We also estimated that ~78% (205 of 262) of the existing *L. cuprina *nucleotide sequences in Genbank were represented in our EST collection. Our *Lucilia *ESTs matched 3,280 unique *D. melanogaster *genes (from 3,409 unique polypeptides) at E-value ≤ 1e^-10^; this is equivalent to ~24.1% of the total gene count in *D. melanogaster *(assuming the total number of genes is ~13,600).

### Identification of potential detoxification and insecticide target genes

We identified 12 cytochrome P450 (*Cyp12a5, Cyp12d1, Cyp302a1 *or *disembodied, Cyp307a1 *or *spook, Cyp317a1, Cyp4d2, Cyp4g15, Cyp6a13, Cyp6d2, Cyp6d4, Cyp6d5*, and *Cyp9f2*), 2 glutathione S-transferase (*GstD1 and GstS1*), and 6 esterase (*Glt, Nrt, CG9289, alpha-Est5, CG9287*, and *alpha-Est7*) homologs in the *L. cuprina *(Table 3). In addition to these detoxification gene families, we also identified ESTs encoding target proteins that have previously been implicated in insecticide resistance (see [22-25]). These included homologs of the gamma-aminobutyric acid receptor-associated protein (GABA(A) receptor-associated protein; EST = GI: 333428695), glutamate receptor ionotropic kainate 2 (glutamate receptor 6; EST = GI: 333421827), and a probable sodium channel protein type 9 subunit alpha (EST = GI:333416352).

**Table 3:** Identification of potential detoxification genes in *L. cuprina*

*D. melanogaster *P450*, GST* or esterase genes	Representative *L. cuprina *EST (GI number)
**Cyp12a5**	333435397
**Cyp12d1**	333437090
**Cyp302a1(disembodied)**	333440339
**Cyp307a1 (spook)**	333412120
**Cyp317a1**	333429047
**Cyp4d2**	333426288
**Cyp4g15**	333425740
**Cyp6a13**	333435119
**Cyp6d2**	333415889
**Cyp6d4**	333432766
**Cyp6d5**	333432767
**Cyp9f2**	333420943
	
**GstD1**	333426230
**GstS1**	333429052
	
**Glt**	333415214
**Nrt**	333422289
**CG9289**	333425114
**alpha-Est5**	333429048
**CG9287**	333437990
**alpha-Est7**	333438330

*P450 (cytochrome P450); GST (gluathione S transferase).

### Blast negatives in EST assembly

As of July 2010, 2,207 of the 7,464 unique gene clusters did not match any sequences in the public databases. Three hundred and sixty-five (or 16.5%) of these blast negatives had an ORF (minimum 20 codons), and the average length of their hypothetical polypeptide products was 126 amino acids (median = 126 amino acids; range = 20-584 amino acids) (details, see Additional file 7).

### Anchor loci development and chromosomal synteny in higher Diptera

**Figure 4:**
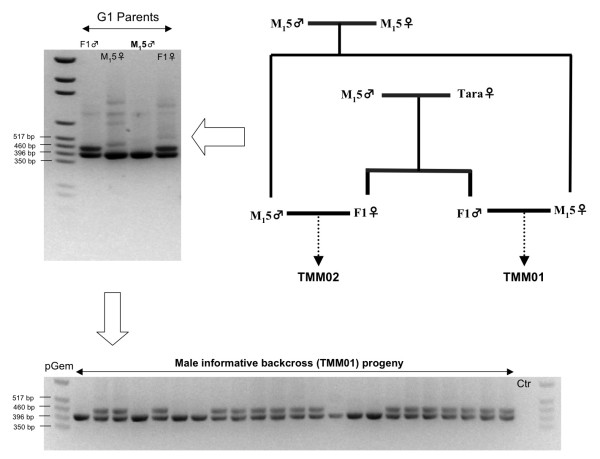
**Mapping anchor loci by scoring intron length polymorphisms**. Top right: Mapping pedigrees were generated using a backcross design initiated using the laboratory strain M_I_5 and the field strain Tara. Top left: Primers were first tested in the 4 backcross parents to identify intron length polymorphism. Bottom: Informative primer pairs were used to screen the backcross pedigree (TMM01).

**Figure 5:**
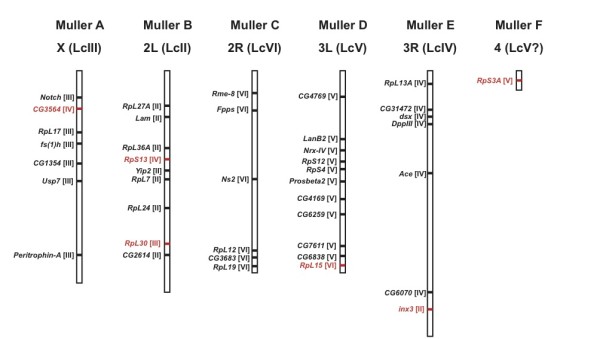
**Chromosomal synteny between *L. cuprina *and *D. Melanogaster***. Genomic locations of the anchor loci are shown on the 6 *D. melanogaster *chromosome arms (X, 2L, 2R, 3L, 3R, and 4). The corresponding chromosomal origins (II, III, IV, V, and VI) of the *Lucilia *homologs are indicated in brackets. Six instances of synteny violation are shown in red.

One aim of the current EST project was to identify single-copy genes that are highly conserved between species for synteny comparison. Reciprocal homology searches among *L. cuprina, D. melanogaster*, and *A. gambiae *yielded a set of reciprocal best-hit trios, and 298 of such trios had favorable intron position and size range in *D. melanogaster *(Additional file 8). This list of 298 orthologous groups facilitated our ongoing linkage map construction in *L. cuprina*. We successfully assigned 41 gene markers to 5 linkage groups using a male informative pedigree by scoring intron length polymorphisms (Figure 4). Comparison between *L. cuprina *and *D. melanogaster *based on 41 gene markers revealed a high level of synteny, although several deviations were also evident (Figure 5; Additional file 9). Deviations included *inx3 RpL30, CG3564, RpS13*, and *RpL15*., A potential translocation or fusion/dissociation event was identified between the smallest chromosome (Muller F in *Drosophila*) and an autosome (Muller D in *Lucilia*), as suggested by the location of *RpS3A *(Figure 5).

## Discussion

The main outcome of this project is the significant improvement of the gene inventory for the Australian sheep blowfly *Lucilia cuprina*. Amongst other applications, this new resource presents promising benefits to such areas as medical, forensic, pest control, and the understanding of genetic adaptation to insecticides.

Barring major gene expansion or contraction, and assuming that *L. cuprina *has the same number of genes as in *D. melanogaster *(~13,600) [26], the 7,464 unique gene clusters we found in our EST libraries would account for up to 55% of the genes present in the species. The actual percentage is much lower due to (but not limited to) the TGICL assembly parameters and the presence of 4.7% short (≤ 100 bases) sequences (Figure 2). An estimate of 24.1% gene coverage was obtained by limiting homology comparison to *L. cuprina *and *D. melanogaster*. However, fast-evolving genes and gene families that have been expanded in the blowfly lineage are under-represented in this analysis. Hence, the estimate of 24.1% could be considered the lower bound of total gene coverage. Nonetheless, this is a conservative yet reasonable estimation given that our cDNA libraries were not experimentally normalized and that only preadult developmental stages contributed to the transcript pool. The EST sequences contain a large number of recognizable protein motifs, as suggested by InterProScan results (Additional file 5), whose protein products are likely to participate in a myriad of biological and cellular processes, as also suggested by Gene Ontology analysis (Additional file 6).

Compared to *D. melanogaster, L. cuprina *appears to have low GC content and a different codon preference for many amino acids. Despite the fact that the comparison was based on 200 conserved gene homologs, the codon preferences for *D. melanogaster *are consistent with those reported by Vicario et al. [27]. The higher effective Nc in *L. cuprina *(43.81) than *D. melanogaster *(40.89) suggests a weaker selection constraint on codon usage in *L. cuprina*, at least for these highly conserved genes. It is noted that the 200 sequence pairs analyzed represent only a small fraction (1.5%) of the coding sequences in the 2 species; perhaps a different pattern might emerge when less-conserved gene homologs are included. Nevertheless, these results could be useful for training gene-finding algorithms and the analysis of the full genome sequence when it becomes available.

The acquisition of > 3,280 blowfly genes allows more sophisticated experimental systems to be developed in the future. Aside from the improvement in the knowledge about the genetic composition of the species, the dataset provides a foundation for designing gene-based microarrays for expression profiling. Furthermore, the plasmid collections can also serve as a permanent source of cDNA clones for protein expression, *in situ *hybridization, and even for transgenic manipulation such as those described in [28-30]. The sequence knowledge of the housekeeping genes such as the ribosomal protein genes, tubulin, and actin could serve as internal controls for quantitative real-time PCR. In fact, the need for such reference genes was recently discussed in [31]. The availability of the *L. cuprina *cDNA sequences would also facilitate quantification of expression profiles of many genes of interest, bypassing the time-consuming gene discovery steps. It is expected that our EST collection will be invaluable for annotating the genic regions of the *L. cuprina *genome, when it is eventually sequenced. Conversely, the cDNA information could itself serve as a gene database, such that short peptides generated by the high-throughput proteome sequencing, similar to those reported in the brain tissues of another blowfly, *Protophormia terraenovae *[32], could be compared, forming a transcriptomic-proteomic feed-forward loop.

We identified genes that are related to insecticide resistance in *L. cuprina *(Table 3). Isolation of these homologs in *L. cuprina *would allow their expression patterns to be accurately measured (e.g., by real-time PCR), and their roles in insecticide resistance to be evaluated. PCR assays to screen for naturally occurring DNA polymorphisms (e.g., exon-primed intron-crossing (EPIC) markers) could also be developed to monitor the temporal and spatial distribution of different alleles. While many of their *D. melanogaster *homologs have been implicated in insecticide detoxification [33-36], some of the genes identified are involved in other developmental processes such as ecdysone biosynthesis (*disembodied *and *spook*) [37,38] and brain function/development (*Cyp4g15*) [39]. The proportions of the new *L. cuprina *homologs represent only a small fraction of these 3 detoxification gene families (see [40-42]). With the advent of next-generation sequencing (NGS) technologies, large-scale genome or transcriptome sequencing has become increasingly popular. For example, transcriptomic analyses using NGS have now been reported in many non-model insect species [43-48]. Similar approaches could be extended to *L. cuprina *and other related blowfly species, to enable a more comprehensive assessment of novel insecticide targets.

Another important application of our newly identified ESTs was to improve the genetic map of *L. cuprina*. ESTs can be converted to a set of anchor loci for linkage mapping, as has been repeatedly shown in other insects [49,50]. We adopted a conservative "reciprocal best hit with strong homology" strategy in the selection of homologous markers, in which *D. melanogaster *served as the primary reference. *A. gambiae*, which diverged from the *Lucilia *and *Drosophila *lineages about 250 MYA, acted as an outgroup to improve the confidence in orthology calling, as sequence homology needed to reach the E-value of ≤ 1e^-50 ^threshold to be considered further. In other words, we opted for confidence rather than sensitivity in our search for orthologous markers.

The EST-derived markers constituted a substantial proportion of anchor loci in the present study and were useful for inferring chromosomal synteny (Figure 5). Linkage assignment of 41 markers allowed us to conclude that chromosomal synteny is high between the *Lucilia *and *Drosophila *lineages. Our results are typical for higher dipteran species, as suggested by previous studies [17,51,52]. Several chromosomal fusion/dissociation events have nonetheless been documented within the *Drosophila *genus. For example, the fusion of Chr 4 (Muller's element F) to an autosome was found in *Drosophila willistoni *[53]. Moreover, comparison between mosquito and *Drosophila *reveals that patches of syntenic regions are scattered across many chromosome regions [54]. Our mapping results suggested that gene content on each of the Muller's elements in *L. cuprina *can, to a large extent, be predicted from the *D. melanogaster *map. However, the obvious cases of synteny violation (Figure 5; Additional file 9) would mean that direct extrapolation of linkage information from *D. melanogaster *would require extra caution. The interspecies differences should justify future *de novo *construction of linkage maps for *L. cuprina*, with denser markers.

The 298 putative orthologs effectively form a pipeline for future comparative mapping efforts (Additional file 8). Their chromosome addresses in *D. melanogaster *span virtually all regions of the genome, allowing flexible control over marker density for genomic regions of interest. Several chromosomal areas in *Lucilia *are of significant historical importance: the *Scallop/Notch *[55,56] on chromosome II and the *Rop-1 *[12,57] regions on chromosome IV. In fact, several gene markers generated in the present study have already been utilized to understand the patterns of selective sweeps around the *Rop-1 *locus [58]. The marker pipeline also offers a starting point for fine scale mapping of the fitness modifier locus (*M*), which is believed to counter the fitness disadvantage of the diazinon-resistant flies in the absence of insecticide [59-61]. Together with an appropriate genomic library, these newly acquired ESTs provide an ample supply of markers for positional cloning of the *M *locus.

The evolutionary origin and phylogeny relationship among blowfly species has been of great interest to many researchers, owing to its medical and forensic implications [62,63]. With the much expanded gene repertoire, some of the *L. cuprina *genes identified here, especially those that show least similarity to other known sequences could be utilized to develop species diagnostic assays. The current EST sequences would greatly complement such an exploration.

While the assemblage of 29,816 ESTs into 7,464 was straight forward, the interpretation of the information contents requires regular re-adjustment, in light of the constantly expanding sequence databases in other species. In order to evaluate the coding components of the newly acquired sequences, they were sorted according to the level of homology to their counterparts in the Genbank reference protein database, producing a typical BLAST significance spectrum (Figure 1). It is anticipated that such a spectrum would change over time. As new sequences from other organisms become publicly accessible, it would simultaneously alter the structure of the existing sequence databases and hence the BLAST results. The recently released EST collections (116,737 reads) from 3 closely related taxa (Glossina, Cochliomyia and, Muscinae) clearly illustrate this notion (Figure 1).

Given that the number of non-redundant sequence clusters depends largely on the assembly settings, the "90% identity over 50 bases" requirement could be viewed as a balanced option, but might not be an optimized condition for all genes. One indication is the presence of residual sequence redundancy in the dataset, presumably due to the natural existence of splice variants, transcript isoforms, natural polymorphisms, or genuine gene families. Hence, it is worthwhile to disassemble relevant contigs that belong to the gene of interest and find the most appropriate parameters to reassemble these reads. Furthermore, we did not impose any restriction on the length of the sequences, i.e., removal of assembled contigs or reads less than a certain length (e.g., 200 bases), because such sequences could be part of the untranslated regions of many legitimate mRNA transcripts. As more similar EST sequences from closely related taxa become available, these short reads might ultimately be informative in the future. In summary, the TGICL assembly described in this paper only represents a generic, non-discriminatory clustering approach for the entire dataset, and re-assembling for the original ESTs might be necessary to produce the most accurate assembly for a given gene or a set of related genes.

## Conclusions

We report the generation of 29,816 ESTs (7,464 unique clusters) from the Australian sheep blowfly *Lucilia cuprina*. Homology analyses revealed that the dataset captured a wide diversity of genes, including those related to insecticide resistance targets and detoxification gene families. Our data also indicate that *L*. cuprina coding sequences are AT rich and that codon usage patterns are distinct from that of *D. melanogaster*. In addition, a subset of putative orthologous genes was identified and mapped to the *Lucilia *linkage groups, which revealed a high but incomplete chromosomal synteny with *D. melanogaster*.

## Methods

### Construction and sequencing of cDNA libraries

Three cDNA libraries were constructed using RNA extracted from embryos, first-instar, and third-instar larvae. Construction of the embryonic cDNA library was previously described in Chen et al. (1998) [55]. Embryonic RNA was extracted from the non-modifier "seeking" strain using the Gibco-BRL mRNA Isolation System (Gaithersburg, MD). A unidirectional cDNA library was constructed in the *Eco*RI/*Xho*I sites of the λZAP II vector (Stratagene, La Jolla, CA), and sequenced from the 5' end using the T3 primer and the 3' end using the T7 primer. Two larval cDNA libraries (first and third instar) were made using total RNA from the inbred laboratory M_1_5 strain. cDNA was prepared using the SMART^® ^cDNA library construction kit (Clontech Laboratories, Inc.), directionally cloned into the αTripIEx2 vector via the *Sfi*-I A/*Sfi*-I B restriction sites, and transformed into BM25.8 competent cells. Plasmid cDNA clones were sequenced from the 5' end using the Sp6 primer. All sequencing was completed using the Sanger dideoxy sequencing method at the Australian Genome Research Facility (AGRF) in Brisbane, Australia. Original EST reads were subject to standard quality-trimming, vector-removal, and poly-A-clipping procedures. The output sequences were then assembled using the TGICL (TIGR Gene Indices clustering tools) algorithm [64] with the minimum threshold level set at 90% identity over a stretch of 50 bases.

### Homology comparison

The GenBank non-redundant reference protein database (8,328,903 sequences) was downloaded from ftp://ftp.ncbi.nlm.nih.gov/blast/db/ in July 2010. The complete *L. sericata *mitochondrial genome (GI:154623433) [21] was also retrieved from Genbank. The 18,648 EST sequences from *C. hominivorax *(primary screwworm), 18, 797 EST sequences (generated by 454 GS FLX; SRA: SRA012250) from *Stomoxys calcitrans *(the stable fly), and 79,292 EST sequences from *G. morsitans *(tsetse fly) were batch downloaded from GenBank via the species taxonomy page in the National Center for Biotechnology Information (NCBI) http://www.ncbi.nlm.nih.gov/taxonomy/. The *D. melanogaster *and *Anopheles gambiae *proteome sequences were retrieved from FlyBase http://flybase.org/ and VectorBase http://www.vectorbase.org/, respectively. All sequences were converted into separate local databases using the NCBI standalone BLAST executables. Homology searches (BLASTX) were performed with E-value cut-off at 1e^-10^. To estimate gene coverage of our *Lucilia *dataset, we performed a BLASTX (E-value ≤ 1e^-10^) search against the *D. melanogaster *peptide database (r5.37). Due to the existence of isoforms in the BLASTX hit list, we extracted their corresponding gene identifiers (i.e., CG numbers and gene symbols) from FlyBase to estimate the number of unique genes. To identify putative orthologs among *Lucilia, Drosophila *and *Anopheles*, a more stringent BLAST E-value threshold (1e^-50^) was used to retain only the most conserved homolog pairs. EST sequences that failed to find a significant match in the reference protein database were then used to search (BLASTN) against the *L. sericata *mitochondrial genome, the *C. hominivorax *and the *G. morsitans *sequences with E-value cutoff at 1e^-10^. Finally, a TBLASTX search (at E-value < 1e^-10^) was performed for EST sequences that did not find sequence homology in both the BLASTX and BLASTN searches.

### GC content and codon bias analyses

To compare the GC content and codon usage properties between *L. cuprina *and *D. melanogaster*, we confined our analyses to 200 homologous sequence pairs. These 200 homologous pairs are highly conserved (BLASTX E-value < 1e^-50^) at the amino acid level and have identifiable complete open reading frames (ORFs) (see Additional file 3). Putative ORFs in *Lucilia *were extracted using the GENSCAN program developed by Burge and Karlin [65]. The homologous ORFs (or CDS) in *D. melanogaster *were retrieved from FlyBase http://flybase.org/static_pages/downloads/ID.html. GC content and codon usage statistics were calculated using the GEECEE program http://emboss.sourceforge.net/apps/cvs/emboss/apps/geecee.html. The effective Nc was estimated using the CHIP program http://emboss.sourceforge.net/apps/release/5.0/emboss/apps/chips.html. All 3 programs were available at BioManager http://biomanager.info/ maintained by Peter Reeves at the University of Sydney, Australia.

### Pedigree construction

The M_1_5 and the Tara strains were used to generate the male and female informative mapping families. The M_1_5 strain is highly inbred and carries 1 visible phenotypic marker on each of its 5 autosomes. The Tara strain (provided by Garry Levot) is a more recent field-derived strain that originated from Tara, Queensland, Australia. It is morphologically wild type and displays high levels of resistance to diflubenzuron and tolerance to cyromazine. To generate the male informative family TMM1, an F1 male from a single pair mating between an M_1_5 male and a Tara female was backcrossed to a virgin M_1_5 female (Figure 4).

### Choice of markers

cDNA sequences (previously characterized genes and ESTs) were converted into gene markers for linkage analysis and synteny comparison. Marker selection was based on (1) their physical locations in *D. melanogaster*, to ensure an even coverage of all Muller's elements; (2) that these genes contain intron(s) of suitable size (100-400 bases); and (3) that the intron positions are conserved in both *D. melanogaster *and *A. gambiae*. EPIC primers were designed using the Primer3 program http://frodo.wi.mit.edu/primer3/ (see Additional file 9 for primer information).

### DNA isolation, polymerase chain reactions, and electrophoresis

DNA from all individuals in the pedigrees was extracted using DNAzol^® ^reagent (Invitrogen; Cat. No. 10503027). In subsequent genotyping assays, 0.1% of the whole body DNA in 1 μL was used per PCR reaction. PCR was done in 25 μL reactions, which contained 1 μL of genomic DNA, 2.5 μL of 10 × reaction buffer, 3.0 μL of MgCl_2 _at 25 mM, 2.5 μL of dNTPs at 2 mM, 1 μL of each of the forward and reverse primer at 10 μL, 0.3 μL of *Taq *DNA polymerase (Fermentas; Cat. No. EP0402), and 13.7 μL of nuclease-free water. We used a touchdown thermo-cycling strategy for all PCR amplification, which involved an initial denaturation step at 95°C for 5 min, followed by 30 cycles of 95°C for 30 s, 65°C for 30 s (reduce 0.5°C per cycle), and 72°C for 2 min, followed by another 30 cycles of 95°C for 30 s, 50°C for 30 s, and 72°C for 2 min. PCR amplicons were separated by electrophoresis.

The parents of the mapping crosses were first screened using EPIC primers for detectable size polymorphisms on a 1.2% agarose gel, which contained 1% agarose (Bioline; Cat. No. BIO-41025) and 0.2% Ultra-High Resolution Agarose (Scientifix; Cat. No. 9030A), at 250 V for 25 min. However, if the agarose electrophoresis did not reveal intron size polymorphism, the PCR amplicons were heat denatured (95°C for 3 min) and run on a 6% polyacrylamide gel (SequaGel^®^-6 system, National Diagnostics; Cat. No. EC-836 and EC-841) at 500 V for 1.25 h using the Gel-Scan 2000 system (Corbett Research). The polyacrylamide gels were stained with 1 × SYBRGold^® ^I nucleic acid gel stain (Invitrogen; Cat. No. S-11494) to look for heteroduplex or single-strand conformation polymorphisms. If polymorphism was found in the parents of the mapping family, then identical procedures were applied to genotype the progeny.

### Linkage analysis

Due to the general lack of meiotic crossing overs in dipteran males, markers on the same chromosome are transmitted together from the male parent to its progeny. Under our backcrossing schemes (Figure 4), all polymorphisms should have come from the Tara strain. Hence, the presence or absence of the Tara allele in the male informative family indicates the presence or absence of a specific Tara chromosome. Markers were assigned to the same linkage group if they shared identical segregation patterns in the male informative cross TMM1. Twenty-two backcross individuals from TMM1 were used in genotyping assays.

## Authors' contributions

SFL drafted the manuscript. ZC prepared the cDNA libraries and obtained the ESTs. SFL and ZC performed linkage mapping and quality control of the sequence assembly. AM performed the sequence assembly, gene ontology, and InterProScan. RTG performed homology searches. PB designed and supervised the project. All authors have read and approved the final manuscript.

## Supplementary Material

Additional file 1 ***L. cuprina *non-redundant EST clusters**. A sequence file containing 7,464 *L. cuprina *non-redundant EST clusters in FASTA format. Click here for file

Additional file 2 ***L. cuprina *mitochondrial genes**. A sequence file containing consensus sequences of *L. cuprina *mitochondrial genes in FASTA format. Click here for file

Additional file 3 **Input file for GC and codon usage analyses**. A sequence file containing 200 *L. cuprina *open reading frame sequences in FASTA format for GC and codon usage analyses. Click here for file

Additional file 4 **Homology search results**. A table containing BLAST hits of the non-redundant 7,464 *L. cuprina *EST sequences. Click here for file

Additional file 5 **InterProScan results**. Two spreadsheets containing InterProScan terms captured by the *L. cuprina *EST sequences. Click here for file

Additional file 6 **Gene ontology results**. Two spreadsheets containing Gene ontology terms captured by the *L. cuprina *EST sequences. Click here for file

Additional file 7 **BLAST-negative EST clusters with protein coding potential**. A table containing a list of BLAST-negative EST clusters that have a hypothetical ORF of minimum 20 amino acids. Click here for file

Additional file 8 **Potential orthologous genes among *L. cuprina, D. melanogaster *and *A. gambiae***. A table containing accession numbers corresponding to orthologous genes among *L. cuprina, D. melanogaster*, and *A. gambiae*. Click here for file

Additional file 9 **Synteny between *L. cuprina *and *D. melanogaster *and primer information**. A table containing chromosomal locations of *L. cuprina *genes and their corresponding primer sequences. Click here for file
